# Detection Methods for Pine Wilt Disease: A Comprehensive Review

**DOI:** 10.3390/plants13202876

**Published:** 2024-10-14

**Authors:** Sana Tahir, Syed Shaheer Hassan, Lu Yang, Miaomiao Ma, Chenghao Li

**Affiliations:** 1 State Key Laboratory of Tree Genetics and Breeding, Northeast Forestry University, Harbin 150040, China; tsana011@gmail.com (S.T.); 17725070573@163.com (L.Y.); 3m19961201@nefu.edu.cn (M.M.); 2 Heilongjiang Province Key Laboratory of Sustainable Forest Ecosystem Management—Ministry of Education, School of Forestry, Northeast Forestry University, Xiang Fang District, Harbin 150040, China; shaheer_hassan1993@outlook.com

**Keywords:** pine wood nematode (*Bursaphelenchus xylophilus*), *Monochamus vector*, *Monochamus* species, disease diagnostics, DNA-based methods, PCR-based methods, digital-based methods

## Abstract

Pine wilt disease (PWD), caused by the nematode *Bursaphelenchus xylophilus*, is a highly destructive forest disease that necessitates rapid and precise identification for effective management and control. This study evaluates various detection methods for PWD, including morphological diagnosis, molecular techniques, and remote sensing. While traditional methods are economical, they are limited by their inability to detect subtle or early changes and require considerable time and expertise. To overcome these challenges, this study emphasizes advanced molecular approaches such as real-time polymerase chain reaction (RT-PCR), droplet digital PCR (ddPCR), and loop-mediated isothermal amplification (LAMP) coupled with CRISPR/Cas12a, which offer fast and accurate pathogen detection. Additionally, DNA barcoding and microarrays facilitate species identification, and proteomics can provide insights into infection-specific protein signatures. The study also highlights remote sensing technologies, including satellite imagery and unmanned aerial vehicle (UAV)-based hyperspectral analysis, for their capability to monitor PWD by detecting asymptomatic diseases through changes in the spectral signatures of trees. Future research should focus on combining traditional and innovative techniques, refining visual inspection processes, developing rapid and portable diagnostic tools for field application, and exploring the potential of volatile organic compound analysis and machine learning algorithms for early disease detection. Integrating diverse methods and adopting innovative technologies are crucial to effectively control this lethal forest disease.

## 1. Introduction

The pine wood nematode (PWN), scientifically known as *Bursaphelenchus xylophilus*, is a destructive parasite that spreads pine wilt disease (PWD), causing extensive damage to pine trees in forest ecosystems [1]. Trees play a vital role in terrestrial environments [2]. However, PWD has led to widespread devastation [3]. The economic impact of pine tree loss is a significant concern, and the potential for its global expansion is alarming. PWD commonly affects several pine species [4,5]. PWD is a highly destructive disease that rapidly kills pine trees, often within weeks or months of infection. Furthermore, the PWD outbreak has spread to various ecosystems (different types of environment where pine trees grow, such as forests, plantations, and even urban parks), establishing strong infection chains [6]. In forests, invasive plant pests such as diseases and insects are the primary cause of tree damage [7,8]. The State Forestry and Grassland Administration found this nematode in northern China, where temperatures drop below −20 °C. This suggests that the nematode-transmitted sickness could expand to cooler Chinese regions [9].

Approximately USD 1.7 billion is spent annually by the United States government on monitoring and controlling 62 high-impact species [10]. Forest pests can significantly affect the nitrogen cycle and species habitats [11]. In 2018, in Liaoning province in China, Yu et al. identified *Monochamus saltuarius* as a vector for PWD. Since this discovery (in 2019), the lumber industry has suffered substantial financial losses [12,13,14]. Spanning sixteen provinces, it stands as a threat to the pine forests of northern China. The proliferation of PWN relies on the presence of *Monochamus* insects [15,16,17]. When infested beetles feed on the tender shoots of healthy trees, they can transmit the nematode, potentially leading to infection [18]. In 2023, several notable incidents of pine tree decline were recorded, primarily attributed to environmental factors such as drought and pest infestations [19]; this reference describes this disease’s damage.

Recent years have witnessed significant advancements in understanding the specific proteins utilized by PWN to invade and effectively feed on trees [20,21,22]. Infected trees exhibit a marked reduction in resin secretion, resulting in a slowdown or cessation of needle transpiration [23]. Thorough culture and inoculation experiments have unequivocally confirmed *B. xylophilus* as the primary causative agent responsible for PWD [24].

Recently, there has been an increasing emphasis on environmentally sustainable control methods, resulting in considerable attention on biocontrol compounds derived from PWD as safe alternatives for plant protection [25]. Repetition of these nutrients may cause PWNs to develop resistance due to selective pressure. Monitoring resistance patterns and evaluating nematicides is essential to finding cost-effective and environmentally friendly PWD treatments. Researchers have long suggested using plant extracts, essential oils, and volatile chemicals to combat nematodes [26,27].

This thorough review presents the most recent research on preventing PWD, which is spread by the longhorn beetle vector *Monochamus alternatus*. The review data were carefully collected from peer-reviewed journals and trusted websites. This analysis critically evaluates current methods and proposes new ones to improve PWD diagnosis accuracy and speed. Implementing successful management methods quickly requires accurate and fast diagnosis. This paper also assesses current diagnostic methods and examines the possibilities of new technologies in PWD detection research.

## 2. Intervention Method

*M. alternatus*, a longhorn beetle, exhibits high CO_2_ concentrations due to respiration cycles and CO_2_ bursts during the feeding phase of maturation, making it an insect carrier for PWN. The nematode detaches from its insect carrier due to the elevated CO_2_ levels [28]. *M. alternatus* primarily spreads through flight, with adults capable of traveling up to 5 km over their entire life cycle [29]. Researchers have implemented multiple measures to ensure the security of the system, including tree injections, insecticide spraying, and the removal of infected trees. These parameters were selected in collaboration with the most relevant factors, excluding the natural birth and death rates of trees, as the primary objective is tree conservation; the disease can be eradicated by practical measures, except for individuals who are asymptomatic carriers. By incorporating this class, the model demonstrated that the disease has the potential to persist as an endemic condition, necessitating ongoing management measures, even under the most favorable circumstances [30]. After a comprehensive review of the control measures, the study concluded that the removal of infected trees is the most effective approach, especially when combined with insecticide spraying. If the cost of insecticide spraying becomes prohibitively high, tree injection can serve as a partial substitute. However, if the expenses associated with the removal of infected trees increase excessively, there is no readily available alternative, potentially leading to uncontrollable costs.

## 3. Conventional Detection Methods

### 3.1. Vector of PWN

The transmission of PWN from dying pines to susceptible living pines is entirely dependent on vector beetles of the genus *Monochamus.* There are geographical differences in the vector species during the PWN invasion. *Monochamus* carolinensis is the main vector of PWN in North America, whereas it is *M. alternatus* and *M. salturatis* in Asia and *M. galloprovincialis* in Europe, the invasion areas of PWN. The evolutionary ecology of PWN is strongly influenced by the change in vector species. The basic molecular pathway for these vector insects is not yet clear due to limited genetic resources [31,32]. This vector beetle and PWN have established a close symbiosis characterized by their extremely synchronized life cycles supported by chemical indicators. The *M. alternatus* beetles prefer to lay their eggs in weakened or dying trees infected with PWN, which facilitates the rearing of their young. The third-stage (LIII) PWN juveniles are attracted to certain terpenes secreted by the mature insect larvae and congregate around the pupal chambers in infected trees. The fourth-stage (LIV) juveniles are attracted to the trachea of newly hatched adults by ascarosides excreted by beetles [33]. 

The newly hatched beetle feeds on healthy trees as it matures. The nematode then leaves the spiracles, driven by the CO_2_ resulting from feeding behavior, and invades fresh, healthy trees through the feeding wounds, beginning a new cycle of infection, reproduction, and dispersal. Although the chemical signals within the symbiosis have been well defined, the molecular mechanisms of this chemical exchange remain unclear. Furthermore, as with most vector-borne diseases, control of the vectors is essential for effective control [28,34]. Unfortunately, there are still no effective and efficient control methods for this vector beetle. Therefore, a high-quality reference genome is essential for a better understanding of this symbiosis and its conservation, as well as for the development of novel control strategies, such as genetically modified management approaches. Gao et al. assembled the genome of *M. alternatus* at the chromosome level using nanopore sequencing. They used Pacific Biosciences (PacBio) HiFi, high-fidelity (HiFi), high-throughput chromosome conformation capture (Hi-C), and Illumina short-read sequencing. The team proceeded with the structural and functional annotation of the assembled genome by integrating transcriptome data from different tissues of *M. alternatus*. The reference genome of *M. alternatus* is a valuable resource for studying the evolution of coleopteran insects and the interaction mechanisms between the PWN and its vector insects [32].

### 3.2. Morphological Identification Visual Inspection

**Figure 1:**
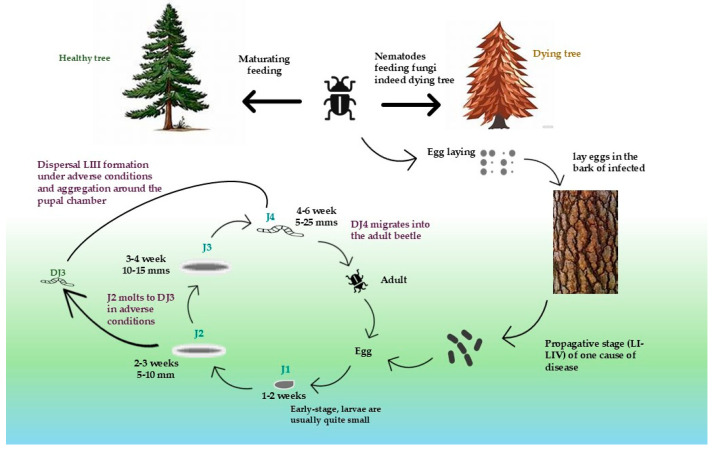
PWN undergoes a multistage lifecycle, commencing as an egg and progressing through four distinct larval phases (L1 to L4) before ultimately maturing into an adult. Under optimal environmental conditions, these nematodes possess the capability to complete their lifecycle in as few as 4 to 5 days. They rapidly spread to new host trees via their primary vector, the *Monochamus* beetle, and reproduce within the host tree while primarily feeding on its vascular tissues.

To comprehensively understand the behavior of *M. saltuarius* and develop effective control measures, additional information on its ecology, pheromones, and potential management strategies is essential [35,36]. Nematodes can infest trees through two mechanisms: a dispersive cycle involving insect vectors or a propagative cycle without insect assistance [37]. PWN exhibits two distinct stages: a phytophagous phase, during which the nematodes feed on live host tree cells, and a *mycophagous* phase, where they consume fungi within the tree, with both contributing to the tree’s decline [38]. These stages manifest differences between male and female adults and four juvenile stages (found in eggs, as J2, J3, and J4) that facilitate reproduction [39]. As shown in Figure 1, nematode stages are J2, J3, and J4, and JIII–JIV stages are characterized by a thick protective cuticle, a distinct head region, a rounded tail end, and the accumulation of lipid droplets in its intestine. These organisms lack an esophagus, stylet, and esophageal glands, and their cuticle’s sticky coating facilitates transport by insect vectors. The tail is relatively cylindrical with a digitate (finger-like) terminal [40]. As winter ends, the nematode enters a hibernation state within *Monochamus* beetles. Once the adult beetles emerge from the tree and begin feeding on healthy twigs, the females lay eggs on damaged trees, allowing the nematodes to continue their life cycle [41].

After infection, the top half of the tree may dry up, and severe cases may exhibit weak branches. These symptoms become most apparent in late summer and early fall when water and nutrient transport within the tree decreases. The severity of the symptoms can vary based on factors such as the tree species, temperature, and time of year [42]. Infected trees may release and accumulate ethylene and condensed tannins (proanthocyanidins), resulting in tissue browning, presence of superoxide anions, changes in ray parenchyma cell vacuoles, lipid peroxidation, electrolyte leakage, release of volatile compounds, and accumulation of phytotoxic substances [43]. Ma et al. conducted an experiment observing resin secretion from pine trees by creating a 10–15 mm spherical hole in the tree trunk’s xylem at chest level. They identified five distinct phases of resin flow: 1. significant exudate and resin outflow from the hole in the primary flow; 2. resin outflow and some exudate in the secondary flow; 3. resin depositing on the hole’s lower edge; 4. granular resin releasing from the hole wall; and 5. no resin flow. Their study indicated that the presence of resin deposited on the lower edge of the hole suggests a potential PWD infection [44]. Some researchers propose that PWN may produce phytotoxins that induce host cell death, possibly explaining the rapid onset of symptoms and cell death in certain cases [45]. Several investigations have suggested that these toxins may have a bacterial origin [46]. However, the accuracy of this diagnostic method can be influenced by various factors affecting pine tree resin flow. Pine trees become particularly challenging to treat once resin flow occurs, typically in the fifth disease stage. Consequently, this approach is primarily used for supplementary diagnosis rather than as a primary diagnostic tool. The traditional method for detecting PWN involves morphological identification of the pathogen *B. xylophilus*. However, molecular diagnostic methods offer the advantages of being faster and more accurate.

### 3.3. Visual Inspection

**Figure 2:**
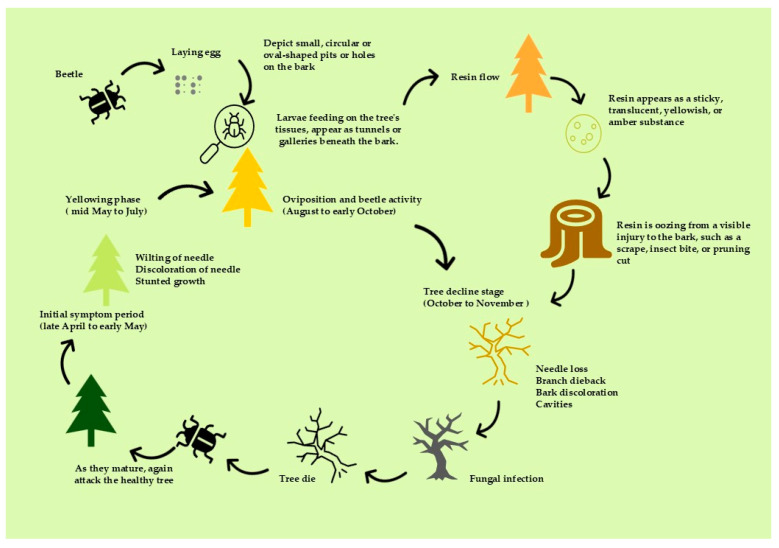
Disease progression typically involves a series of stages that delineate the advancement and worsening of a condition over time. In the context of infectious diseases, these stages include the incubation, prodromal, acute, and convalescence periods. Each stage is characterized by distinct symptoms and physiological alterations that influence the strategies employed for treatment and management.

Liu et al. (2019) state that the typical onset of the condition occurs between April and November. The needles remain attached to the tree as they lose their vibrancy, transitioning in color from green to yellow and ultimately turning reddish brown. Beetle egg-laying sites are commonly found on the bark. As illustrated in Figure 2, scraping off a small piece of bark does not result in the release of any resin [47].

During the spring and early summer months following a PWD infection, the needles of infected pine trees exhibit similarities to those of winter-damaged pines. Removing the bark will result in resin leakage. In contrast to healthy pine trees, where needles discolor due to dryness, the needles of PWD-infected trees begin to change color at the base and progress upward. This approach is useful for surveying diseases in extensive forests, but it necessitates trained professionals with knowledge of the local forest environment and the ability to identify signs of pests, insects, and plant diseases. The method can serve as an initial assessment of PWD, but it should be supplemented by a pathogen identification procedure to ensure accuracy and applicability. Furthermore, other factors that may contribute to tree damage, such as tree age, soil health, and the extent of environmental damage, should be considered. Young trees are more susceptible to damage but possess the ability to recover within the same season [47].

## 4. DNA-Based Detection Methods

**Figure 3:**
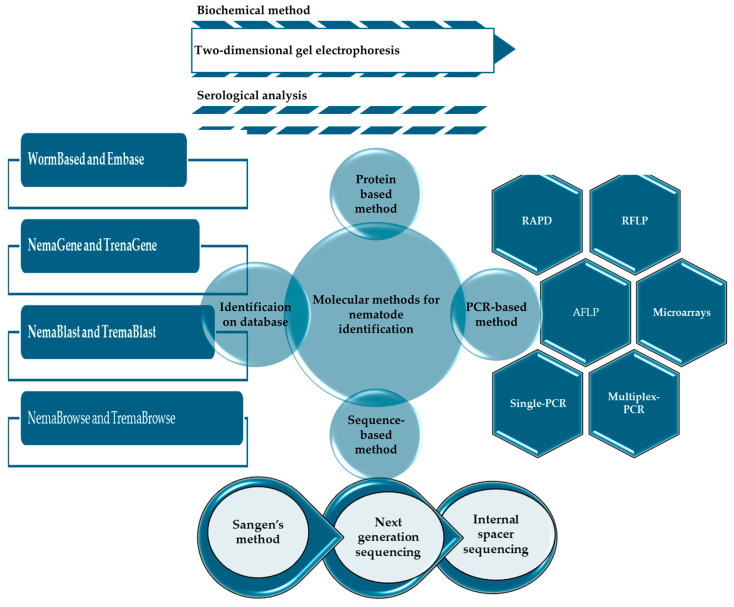
The techniques employed for nematode protein identification have the potential to significantly enhance the accuracy and efficiency of diagnostic procedures for species such as *B. xylophilus* and *B. mucronatus*. These advancements could substantially impact pest management strategies and ecological research. However, the labor-intensive nature of these techniques and the necessity for a positive reference sample may limit their practical application in rapid field assessments and routine monitoring.

Relying solely on a specimen’s physical characteristics for diagnosis can be prone to errors due to subjective human judgment. In contrast, scientists have developed advanced molecular detection methods that specifically target the DNA of PWN [48]. Recent advancements in the wood market have surpassed traditional morphological identification methods. DNA technology has revolutionized the development and enhancement of molecular detection techniques for PWN, significantly expanding their practical applications [49]. Various DNA-based approaches have been developed over time to detect nematodes. These methods often involve analyzing distinct genetic markers or sequences unique to nematode species. By utilizing DNA sequencing and PCR, researchers can accurately identify and detect nematodes in various environments. These technologies have transformed the study of nematodes by providing rapid, precise, and sensitive methods for species identification, leading to substantial advancements in understanding their ecology, biodiversity, and interactions with other organisms [50,51,52,53]. Techniques for identifying nematodes can be broadly categorized as either fingerprint-based or nucleotide-based. Fingerprinting methods, except RFLP, rely on PCR amplification and electrophoresis to generate a DNA fingerprint. This category includes AFLP, RAPD, RFLP, and species-specific primers. Subsequently, phylogenetic analysis and nematode identification are performed based on this genetic template. In contrast, nucleotide-centric techniques involve sequencing DNA segments for phylogenetic evaluation and employing probes for hybridization and PCR amplification. Each method of worm identification has its advantages and disadvantages, and it is crucial to emphasize the significant contribution of nematode sequences in advancing the understanding of evolutionary relationships among species [49]. The following excerpt explores DNA-based molecular detection technologies, thoroughly assessing their technical attributes and potential applications in the field of motor vehicle identification. The primary objective is to gather comprehensive information to support the development of advanced and efficient molecular detection methods essential for PWN management. The rapid advancement of PCR has greatly facilitated the extensive identification and categorization of PWN. Since its introduction, PCR has been fundamental in molecular biology. Currently, standard PCR detection methods for PWN include RT-qPCR, DNA barcoding, ITS-RFLP, SCAR, and RAPD [54]. The utilization of molecular techniques provides a solution to the challenges associated with conventional morphological identification methods. PCR assays enable the accurate identification of multiple nematode species in a mixed sample. This sophisticated approach not only reduces the time required for diagnosis but also decreases associated costs [55]. The previously outlined methods are effective in identifying nematodes and enable prompt diagnostic testing. By combining CRISPR Cas12a with recombinase polymerase amplification (RPA) and LAMP procedures, it is possible to achieve exceptionally accurate detection capabilities [56]. DNA-based detection methods have revolutionized the techniques for identifying nematode populations, as illustrated in Figure 3.

### 4.1. RPA Method

Recombinase polymerase amplification (RPA) is an isothermal method that is both sensitive and selective, capable of amplifying 1–10 DNA target copies in under 20 min at temperatures ranging from 37–42 °C with minimal sample preparation. RPA has been successfully employed to amplify RNA, miRNA, ssDNA, and dsDNA from various organisms and sample types. The number of publications discussing RPA is increasing, and the technique has been applied in solution, solid, and bridge amplification formats. RPA has been integrated with end-point lateral flow strips, real-time fluorescence detection, and other methods. This evaluation examines the techniques, advancements, advantages, and limitations of RPA technology [57]. Cha et al. [58], for instance, developed the RPA amplification method and primers targeting the 5S rDNA of *B. xylophilus*. This approach can exponentially amplify nucleic acids within 10 min and detect as little as 1.6 pg of genomic DNA from *B. xylophilus* [59]. It demonstrates superior sensitivity compared to PCR and LAMP. However, the simultaneous detection of *B. xylophilus* and *B. mucronatus* has not been achieved previously. Fang et al. designed duplex-RPA primers targeting the ITS regions of *B. xylophilus* and *B. mucronatus*. These primers enabled the rapid and concurrent detection of *B. xylophilus* and *B. mucronatus* by amplifying target nucleic acids within 30 min at 37 °C [60]. The point-of-care diagnostic (POCD)-RPA test is effective in detecting *B. xylophilus*, which facilitates epidemiological investigations and lumber quarantine efforts. RPA technology is user-friendly, even for individuals without extensive nematode expertise. Although nematode identification typically necessitates specialized equipment, the test results obtained through this method are reliable.

### 4.2. RFLPs Method

Restriction fragment length polymorphism (RFLP) analysis can be performed by employing fingerprints generated from genomic DNA (gDNA) digested by one or more endonucleases. Fingerprints can also result from PCR-amplified fragments known as PCR-RFLPs. Although gDNA-RFLPs may be complex, they have the potential to reveal more polymorphisms due to the increased size of the gDNA template. Moreover, gDNA-RFLPs do not require prior sequence information, unlike PCR-RFLPs. Confirming the completion of restriction decisions is crucial to avoid non-reproducible fingerprints [53,61,62]. Previous studies have differentiated *B. xylophilus* and other pine wood nematodes (PWNs) by utilizing the RFLP approach with the 5.8S gene and various restriction endonuclease combinations, such as Dra I and PvuII, Hinf I, Dra I, and Sal I. RFLP technology is essential for evaluating the genetic diversity of nematodes [63]. The RFLP technique is a critical tool for investigating genetic variation among parasite species. It can be used to establish genetic relationships among worm populations and distinguish them. However, this procedure is rigorous and time-consuming, requiring the extraction of highly pure nematode DNA.

### 4.3. RAPD Method

The random amplified polymorphic DNA (RAPD) methodology is an innovative genetic marker method that amplifies target DNA using PCR and a short, random primer of 9–10 nucleotides. Differences in specific DNA bases compared to the primer sequence are utilized to detect genetic variants. Plant parasitic nematodes (PPNs) can be rapidly, effortlessly, and accurately detected using RAPD markers. The RAPD method has been utilized to investigate cyst nematodes and root-knot nematodes. As noted by [54], performing the RAPD assay at low temperatures enhances primer versatility and repeatability among laboratories. These primers attach to distinct locations on the DNA, and amplification occurs when two primers attach to opposite strands of DNA at a distance that allows the polymerase to move across [50]. Employing the RAPD method, Fengmao et al. examined 140 random primers and identified the primer OPK09 and the primer combination OPC18+OPN18 as effective in differentiating between *B. xylophilus* and *B. mucronatus*.

The size of fragments produced in the random amplified polymorphic DNA (RAPD) procedure is significantly influenced by the polymerase enzyme utilized. Therefore, it is crucial to use sufficient quantities of genomic DNA (gDNA). The lower annealing temperatures employed in the RAPD method reduce primer binding stringency, which may lead to issues with repeatability, particularly when the technique is applied across multiple laboratories. Despite this limitation, RAPD offers a significant advantage in that it does not require prior knowledge of the DNA sequence, making it a versatile tool for a wide range of applications [64].

RAPD generates data but necessitates advanced atlases, rigorously controlled experimental reaction systems, and highly sensitive amplification processes. Furthermore, this method must yield consistent and reproducible results across different laboratories. These limitations render the RAPD technique ineffective for the reliable identification of *B. xylophilus*.

### 4.4. AFLP Method

Amplified fragment length polymorphism (AFLP) enhances restriction fragment length polymorphism (RFLP) of genomic DNA by amplifying reduced restriction fragments, resulting in more straightforward fingerprints. This method involves cleaving the genomic DNA using two enzymes, adding adaptors, and amplifying a specific group of fragments using targeted primers [49,65]. AFLP is a cost-effective DNA fingerprinting method that provides valuable information and may be applied to analyze any organism without requiring prior sequence data [66]. A PCR-based molecular approach called amplified fragment length polymorphism, or AFLP, creates and compares distinct fingerprints of genomes by selectively amplifying a fraction of digested DNA fragments from any source. Compared to other widely used DNA markers, it is more efficient in terms of speed, economy, reproducibility, informativeness, resolution, and sensitivity. In addition, relatively little DNA is needed, and no previous knowledge of the genome is desired. Plant taxonomy, genetic diversity, phylogenetic analysis, high-resolution genetic map building, positional cloning of genes, determining cultivar relatedness and varietal identification, and other applications are all made possible by this technology. The paper covers in detail the main benefits and drawbacks of AFLP in plants, as well as its numerous applications. This review also takes into account new advances in polymorphism detection, as well as different variations on this methodology. Additionally, a wet-lab protocol is given [67]. AFLP facilitates the amplification of restriction fragment selective PCR from a whole genomic DNA digest with greater ease. Although AFLP is more repeatable and reliable than RAPD, it may have a longer processing time [68].

AFLP technology has become more user-friendly and accessible due to automation and the availability of commercial kits, despite its sophistication. Although the variability of nuclear and chloroplast sequences may not always be consistent in closely related plant species, the AFLP methodology remains a reliable and powerful technique for accurately identifying the genotypic fingerprint of plants. This is because AFLP markers are widely distributed across the genome, providing a distinct advantage by overcoming the limitations caused by the potential lack of diversity in specific genomic regions. This feature enhances the efficacy of the AFLP approach as a diagnostic and discriminatory tool in plant pathology and molecular characterization [69].

### 4.5. Barcoding Methods

AFLP technology has become more accessible and widely adopted due to automation and commercial kit availability, despite its initial complexity. While nuclear and chloroplast sequence variability may not always be consistent in closely related plant species, the AFLP methodology provides a reliable and powerful approach for accurately identifying the genotypic fingerprint of plants. This is due to its extensive coverage throughout the genome, which gives the AFLP method a unique advantage by overcoming the limitations caused by potential diversity deficiencies in specific genomic regions. This enhances the technique’s effectiveness as a diagnostic and discriminative tool in plant pathology and molecular characterization [70]. DNA barcoding techniques have proven beneficial in examining conspecific relationships and genetic connections in nematodes, particularly in the genera *Meloidogyne*, *Heterodera*, and *Bursaphelenchus* [71,72,73]. A phylogenetic analysis of plant-parasitic nematodes from the family *Longidoridae* utilized mtDNA and rDNA regions, with the study employing 18S rDNA DNA barcoding techniques for community analysis of nematode [74]. However, analyzing eDNA using metabarcoding is linked to several challenges, eDNA is susceptible to contamination during sample extraction and storage. The availability of species-specific DNA barcodes depends significantly on the thoroughness of current databases. Identifying PPN species is challenging because limited DNA barcoding data are available for most identified plant nematodes. DNA barcoding is a promising method with tremendous potential for taxonomic studies, although it faces obstacles [75]. Despite DNA barcoding and sequencing providing powerful tools for identifying and locating the pine wood nematode, numerous challenges must be addressed to fully realize their potential.

### 4.6. Microarray Detection Techniques

A DNA microarray is a collection of minute DNA fragments (picomoles) affixed to specific locations on a solid substrate, such as a glass slide. These DNA fragments, which can be derived from sequence-characterized amplified regions (SCARs), serve as probes to identify nematodes. In high-throughput diagnostic applications, the probes are utilized in hybridization with fluorescently labeled PCR products or genomic DNA (gDNA) from test samples. An array scanner is employed to acquire data from the hybridized slides at the emission wavelengths of the fluorescent dyes [76] and to investigate the potential of *M. Chitwood*-specific DNA microarray probes for nematode identification. The probes were designed using nucleotide sequences within the binding regions of the primer sets employed for the generation of SCAR and satellite DNA fragments in *M. chitwoodi,* but not in *M. arenaria*, *M. javanica*, *M. fallax*, or *M. hapla*. The SCAR-based and satellite DNA-based probes successfully identified *M. chitwoodi* irrespective of the geographical origin of the nematode, demonstrating a specificity comparable to that of the PCR primer sets. Cross-hybridization with *M. chitwoodi* targets was observed when utilizing pMfFd satellite DNA-based probes derived from *M. fallax*, a closely related species, underscoring the importance of judicious probe selection. To date, no other studies have reported the application of DNA microarrays for nematode diagnostics.

TaqMan quantitative PCR (qPCR) is a technique that enables the detection and quantification of nematodes using labeled DNA probes. In TaqMan qPCR, the designated probe hybridizes with the template DNA close to the primers. As the reaction progresses, the polymerase, through its 5′ nuclease activity, cleaves the probe, separating the reporter dye from the quencher at the 3′ end. Each cycle of PCR releases an increasing number of dye molecules, resulting in a proportional increase in fluorescence intensity with amplicon production. The incorporation of probes enhances the specificity of the assay compared to conventional PCR, and the fluorescence signal can be utilized for nematode quantification. Sequence alignments can be employed to design primers and probes for species-specific primers or SCARs. Exemplifying this approach [77], previous studies developed a real-time PCR assay for the detection of *M. hapla* in soil and root galls. The assay specifically amplified *M. hapla* DNA, distinguishing it from 14 other *Meloidogyne* spp. Sequence alignment of the 14 species revealed potential contamination of *M. minor* DNA with *M. hapla*, which was necessary for amplification with the designed primer sets and probe. The assay demonstrated the ability to detect *M. hapla* DNA in as little as 250 mg of soil, equivalent to one-third of an egg. The application of TaqMan qPCR for nematode species detection and quantification has been reported in several other studies [77,78].

### 4.7. Sequence Testing Methods

The method may involve analyzing nucleotide sequence information from specific regions of nuclear DNA, mtDNA, or the entire genome (for gene regions and associated primer sets, see [79,80]. Most studies indicate that the rDNA and mitochondrial COX1 (cytochrome c oxidase subunit 1) genes are optimal for diagnostic purposes [81,82] due to their combination of variable and conserved regions. The variable region of COX1 exhibits significant sequence diversity, making it suitable for resolving issues at lower taxonomic levels, such as species and subspecies groups [83]. In contrast, the flanking regions are highly conserved, facilitating the design of “universal” primers. For instance, a previous study [82] expanded the applicability of rDNA to a wider range of taxonomic levels. The majority of sequence diversity in rDNA is concentrated in the ITS (internal transcribed spacer), which is divided into ITS1 and ITS2, by the 5.8S coding region within the rDNA cistron [84]. ITS is particularly useful for molecular systematics of closely related nematode species [85]. Illumina sequencing, employing short-read ITS-2-rRNA for “nemabiome” metabarcoding, is increasingly used to investigate nematode communities in the gastrointestinal tracts of domestic animals and has been applied to other host species as well [86,87]. In another study [88], soil nematodes were assigned to MOTUs (molecular operational taxonomic units) based on 18S SSU (small subunit) sequence data. Each MOTU comprised a cluster of sequences differing by fewer than three bases, derived from 450–500-nucleotide-long raw sequences generated using primer SSU94, after removing gaps, ambiguous characters, and unresolved base calls. The aligned data ranged from 349 to 396 nucleotides, and MOTU content was inferred from neighbor-joining trees using absolute character differences as distance. The authors observed that MOTUs generally corresponded to morphologically identified species or genera. Additionally, another study [89] investigated the potential of 18S as a barcode for criconematina nematodes. SCAR-PCR (sequence characterized amplified region-PCR) primers have been successfully employed to convert random amplified polymorphic DNA fragments specific to *Meloidogyne incognita* and *M. javanica* into SCAR markers for nematode identification [90] However, the amplification sensitivity in adults was only one-third of that in second-instar larvae. In contrast, Chen et al. achieved accurate larval identification and successfully developed a *B. xylophilus* detection kit using SCAR markers, with a sensitivity one-seventh of that for *B. xylophilus*. The results demonstrate excellent stability and high reproducibility, and the SCAR markers can rapidly detect multiple individuals. However, the detection process requires 2.5–3 h and involves numerous steps [91].

### 4.8. Rapid Detection

This study presents a rapid method for detecting the presence of the PWN, *B. xylophilus*, in preserved *M. alternatus* beetles without requiring a separate nematode extraction process. Wang et al. [92] demonstrated that two PWN-specific primers could effectively distinguish *B. xylophilus* from ten other nematode species, and PCR primers could amplify the DNA of a single nematode in beetle tissue free of nematodes. They also investigated the amplification of a 10-fold dilution series of PWN genomic DNA. To facilitate PCR detection of *B. xylophilus*, an efficient method for preserving *M. alternatus* samples was developed, involving storage in a 75% alcohol solution. The study established an initial protocol for extracting *B. xylophilus* DNA from 2 mg of preserved beetle tissue containing nematodes, enabling their detection through PCR amplification. The PCR assay successfully amplified a 403 bp amplicon (DQ855275) specific to *B. xylophilus* from the preserved beetle tissue. This study represents the first reported instance of rapid detection of *B. xylophilus* in stored *M. alternatus* using rDNA amplification, without the need for a separate nematode extraction step. This finding provides a valuable tool for identifying *B. xylophilus* in preserved *M. alternatus* specimens.

## 5. PCR-Based Detection Method

Since its inception, polymerase chain reaction (PCR) has become an indispensable tool in molecular biology. PCR has transformed fundamental research on the pathogenicity of plant-parasitic nematodes and has revolutionized molecular diagnostics. Molecular diagnosis, employing sequence variants, is a standard method in plant pathology with various applications, such as the study of pathogenic genes, origin identification, and phylogenetic analysis [93]. PCR primers are utilized for pathogen identification, while agarose gel electrophoresis is employed to validate PCR amplification results, enhancing the accuracy of nematode identification. The utility of PCR technology is further augmented when integrated with other molecular biotechnologies, particularly for precise species identification [94,95].

### 5.1. RT-PCR

RT-PCR is a widely used technique for identifying nematode species. RT-PCR can detect *B. xylophilus* DNA at a minimum threshold of 0.005 picograms by employing a specific primer pair and probe targeting the internal transcribed region of the rDNA. Moreover, this method demonstrates sufficient sensitivity to detect even a single *B. xylophilus* specimen [96,97]. RT-PCR offers several advantages, including enhanced sensitivity, safety, reliability, and efficiency. However, the primary drawbacks of this technique are the lengthy experimental process and the high cost of chemicals and equipment. Researchers have developed an improved, highly efficient nematode lysate for RT-PCR, enabling the direct cleavage of nematode DNA from pine material. An automated *B. xylophilus* detection system incorporates output, interpretation, and process design. This innovative approach allows for the detection and analysis of *B. xylophilus* in samples without the need to isolate the nematode from contaminated wood [98].

### 5.2. ddPCR

Droplet digital PCR was initially introduced in 1992 [99] and utilizes the Poisson distribution and single-molecule dilution of templates to facilitate the quantification of DNA molecules [100]. ddPCR involves partitioning a standard PCR reaction mixture, akin to the TaqMan assay, into several small reaction systems. Division can be accomplished by diluting capillaries, oil emulsions, or microtiter plates. ddPCR offers several advantages, including reduced susceptibility to contamination, exceptional precision at low concentrations, and the potential for simplifying sample analysis for diseases that are challenging to diagnose with alternative methods [101,102]. In contrast to qPCR, ddPCR technology allows for the precise quantification of DNA copy numbers. ddPCR is highly sensitive, as it can detect templates in minute quantities without requiring pre-enrichment. This approach has been effectively employed to diagnose infectious diseases in clinical settings. Furthermore, ddPCR has been utilized to detect numerous plant diseases caused by bacteria, viruses, and fungi [103]. Currently, this approach is being applied to detect *Meloidogyne enterolobii* [104].

## 6. PWN Protein Detection Method

Protein-based techniques have gained increasing popularity for nematode identification in recent years. Proteins play crucial roles in various biological processes, including cellular differentiation, proliferation, energy production, and signal transduction [105]. Although proteins have a more limited vocabulary compared to DNA due to genetic code redundancy, their alphabet is substantially more complex, consisting of over 20 characters in contrast to the four DNA bases. The presence of significant species-specific variations in the distribution of membrane proteins enables the identification of different nematode types through protein comparison. An alternative approach to detecting these differences is to analyze the levels and patterns of protein expression [106]. Moreover, protein structure and post-translational modifications contribute to the diversity of nematode species, facilitating the identification of specific nematode species.

### 6.1. Gel Electrophoresis

Researchers have employed the two-dimensional gel electrophoresis (2-DGE) method to study the taxonomy of nematodes [107]. A study of differential gene expression (DGE) results [108] demonstrates that the size and mass/charge ratio of protein molecules can differentiate between various nematode species. To analyze the differences in protein expression patterns between *B. xylophilus* and *B. mucronatus*, we extracted proteins from both species and performed two-dimensional gel electrophoresis. The comparison between *B. mucronatus* and *B. xylophilus* revealed 15 highly expressed proteins [109]. In another study, researchers investigated protein differences in blast lesion mimic (blm) rice mutant leaves from one- and two-week-old plants. They performed gel-based one- and two-dimensional electrophoresis (1- and 2-DGE) on blm and wild-type (WT) leaves before and after lesion formation. The 1-DGE immunoblotting assay showed that BLM mutant leaves had higher levels of the rice phytoalexin sakuranetin enzyme, naringenin 7-O-methyltransferase, and oxidative stress proteins such as ascorbate peroxidase (APX), superoxide dismutase (SOD), and catalase. In 2D gel immunoblotting with anti-APX and anti-SOD antibodies, researchers discovered new blm-cross-reacting protein sites. Fifty distinct Coomassie-blue-stained spots were identified as differentially expressed proteins in BLM using 2-DGE. After removing 23 and 44 protein sites, N-terminal amino acid sequencing and nano-electrospray ionization liquid chromatography–mass spectrometry (nano-ESI-LC-MS) identified 26 non-duplicate proteins. Blm enhanced the expression of disease-related class 5 and 10 proteins and a novel OsPR10d protein. According to the Pro-Q Diamond phosphoprotein gel stain, researchers determined that the osPR5 protein spot was likely a phosphoprotein. Interestingly, leaf OsPR10b protein spot 20 resembled the rice root-specific PR-10 (RSOsPR10) protein. To address this issue, researchers used gene-specific primers and RT-PCR (S1 and S2) to identify RSOsPR10 mRNA in BLM and WT leaves [110].

2-DGE is the only approach capable of rapidly and reliably sorting and displaying hundreds of proteins. The quantity of proteins isolated and the extent of polymorphism observed are dependent on the specific process and sample utilized. Protein spots can be challenging to differentiate, as it may be unclear whether the similarities or differences are inherent or merely a result of gel deformation. Furthermore, certain procedures are more intricate than others, adding to the complexity of the analysis.

### 6.2. Isozyme Method

Many organisms contain isozymes. The most common isozyme separation methods include electrophoresis, chromatography, enzyme tests, and immunology. After extracting soluble proteins from worms, researchers dye specific isozymes. Polyacrylamide gel electrophoresis enables the recognition of nematode species based on the molecular structure, activity, and immunogenicity of isozymes [111]. Morphological features alone can make distinguishing *Bursaphelenchus xylophilus* from other non-pathogenic species challenging. *B. mucronatus* and *B. fraudulentus* bear the closest resemblance to *B. xylophilus*. These nematodes are widespread in Asia and Europe and can colonize pine species. Therefore, phytosanitary purposes necessitate the precise and rapid identification of the three species. In a multiplex PCR, four primers were employed to identify and distinguish the three *Bursaphelenchus* species. The multiplex PCR yielded DNA fragments of 767, 305, and 132 base pairs for *B. xylophilus*, *B. mucronatus*, and *B. fraudulentus*, respectively. This primer combination produced satisfactory results in multiplex PCR experiments with multiple populations of the aforementioned species without cross-reactions with other *Bursaphelenchus* species. The method described offers a simple, reliable, and more cost-effective alternative to previous molecular methods for the simultaneous identification of the three *xylophilus* species. By examining the molecular structure, activity, and immunogenicity of isozymes, polyacrylamide gel electrophoresis can differentiate between various nematode species. A previous study [112] conducted enzyme electrophoresis on *B. xylophilus* and *B. mucronatus*. While differences in malate dehydrogenase, cellulose, and esterase patterns were observed, these differences lacked consistency. Glutamate oxaloacetate transaminase emerged as the sole enzyme capable of distinguishing between *B. xylophilus* and *B. mucronatus*. Currently, researchers employ malate dehydrogenase and superoxide dismutase for nematode protein identification. However, the method could be more efficient and time-consuming, necessitating a positive sample as a benchmark [113].

## 7. Loop-Mediated Isothermal Amplification (LAMP)–CRISPR/Cas12a

Isothermal amplification technologies, such as RPA and LAMP, offer a significant advantage over traditional methods by enabling the identification of *B. xylophilus* without the need for a thermocycling apparatus. The real-time detection capability of these methods makes them particularly suitable for point-of-care and field applications. The simplicity and rapidity of these techniques facilitate prompt detection of *B. xylophilus*, enhancing surveillance efforts and enabling timely treatment of PWD. In regions where access to sophisticated diagnostic equipment is limited, this approach has the potential to improve the identification and accessibility of *B. xylophilus* testing by allowing it to be performed in non-laboratory settings. In summary, isothermal amplification has the capacity to enhance the monitoring and control of *B. xylophilus*, contributing to the effective management of pine wilt epidemics [114,115]. The development of efficient methods for rapid and accurate identification is crucial for the successful management of *B. xylophilus*. Conventional methods rely on expensive equipment and are time-consuming. This advanced technology offers a swift and precise identification of *B. xylophilus*, while being cost-effective and user-friendly, allowing for complete sample preparation, detection, and data processing within one hour. The LAMP-CRISPR/Cas12a assay distinguishes itself from LAMP by employing species-specific fluorescence cleavage to detect target amplicons. This approach reduces the occurrence of false positives arising from non-specific amplicons, validates the authenticity of the amplicons, and eliminates background DNA from the reaction. The LAMP-CRISPR/Cas12a assay successfully detected *B. xylophilus* nematodes in 46 pine wood samples. The data were analyzed using three methods to accommodate different scenarios. The three-step process, consisting of ocular evaluation, lateral biosensor testing, and an integrated molecular analysis system, standardizes and streamlines the detection process, resulting in improved accuracy and efficiency [116]. CRISPR/Cas12a and LAMP have emerged as powerful tools for pathogen identification. The use of Cas12a for virus identification has shown promise and achieved notable success [117]. Moreover, numerous studies have demonstrated the potential of genetic modification using CRISPR to enhance wood quality [118].

Relative expression of pine wood nematodes has been investigated; however, additional research is necessary to determine the optimal and most efficacious approach for integrating diverse detection methodologies.

## 8. Enzyme-Mediated Amplification Method

EMA utilizes special primers and a novel probe targeting the ITS2 internal transcribed rRNA spacer gene. The synergistic action of multiple enzymes enables detection within 40 min, with 10 min for DNA extraction and 30 min for detection. This fast and reliable method enhances the speed and effectiveness of PWN detection, allowing for quick and accurate identification of PWNs from various geographic regions at any age. Furthermore, EMA can distinguish PWNs from other *Bursaphelenchus* species, making it a valuable tool for PWN quarantine testing [119]. The EMA reaction system detects and amplifies fluorescence signals. The amplification process involves helicase, DNA polymerase, single-strand DNA-binding protein, and ATP regeneration protein. Helicases unwind the double-stranded nucleic acid template, while a monomeric binding protein complex with primers precisely binds to the target sequence. DNA polymerase elongates the strand, and the ATP regeneration protein completes the catalysis of the chemical system, amplifying the target region. An endonuclease is required to identify fluorescent signals, cleaving the probe and producing fluorescence to pinpoint the base deletion location [119]. Compared to traditional PCR or morphological testing methods, the EMA methodology provides a more targeted, efficient, accurate, and rapid approach to detecting PWN. This method is poised to be an appropriate and quick diagnostic tool, particularly for users in regions with a higher prevalence of PWD.

## 9. Proteomics

**Figure 4:**
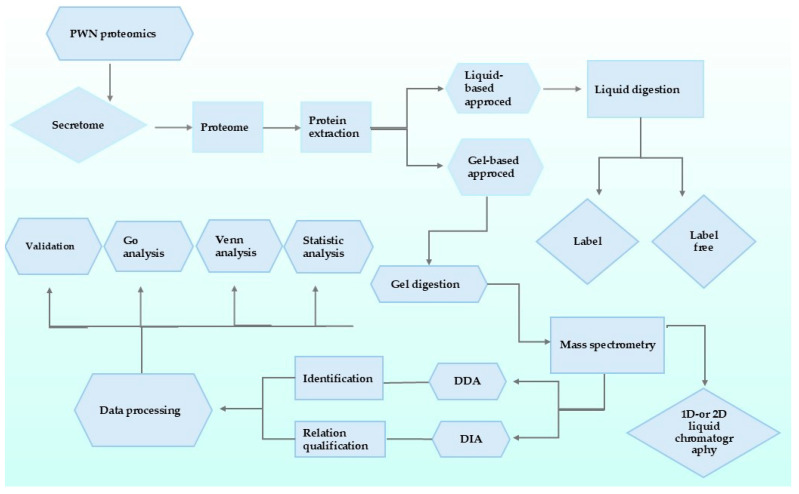
Research on *B. xylophilus* has focused on characterizing its secretome and identifying potential indicators of virulence. Comparative analyses of secretomes and proteomes across multiple *B. xylophilus* isolates reveal variations in protein expression patterns, which may contribute to differences in nematode pathogenicity and host specificity.

Proteomics encompasses a broad array of techniques employed to investigate proteomes, which constitute the entire set of proteins present in an organism or a sample at a specific time point and under distinct conditions [120]. Proteomics methodologies typically initiate by fragmenting proteins into smaller peptides and subsequently utilizing mass spectrometry to detect and identify these peptide sequences. These sequences are then compared to protein sequences stored in databases. The relative quantity of individual proteins in a particular sample is ascertained through a combination of mass spectrometry and bioinformatics [121]. In a recent study, Cardoso et al. employed advanced techniques such as MS (mass spectrometry) and two-dimensional gel electrophoresis (2-DGE) to identify and quantify proteins in *B. xylophilus*. Their analysis examined 1456 proteins from two *B. xylophilus* isolates exhibiting different levels of virulence. The study revealed significant differences in the secreted proteins of the two isolates when exposed to various stimuli from their host organisms [122]. Shinya et al. used advanced nano-LC-MS/MS to identify a remarkable total of 1515 secreted proteins, underscoring the intricate and crucial role of the nematode’s secretome in facilitating its pathogenicity [123]. The *B. xylophilus* secretome comprises proteins involved in diverse essential biological functions, including metabolic processes and cellular activities. They identified proteins with catalytic activities, such as peptidase and hydrolase functions [124]. Mass spectrometry methods, DDA and DIA, analyze complex samples, as shown in Figure 4.

## 10. Remote Sensing

### 10.1. Satellite Detecting Method

**Figure 5:**
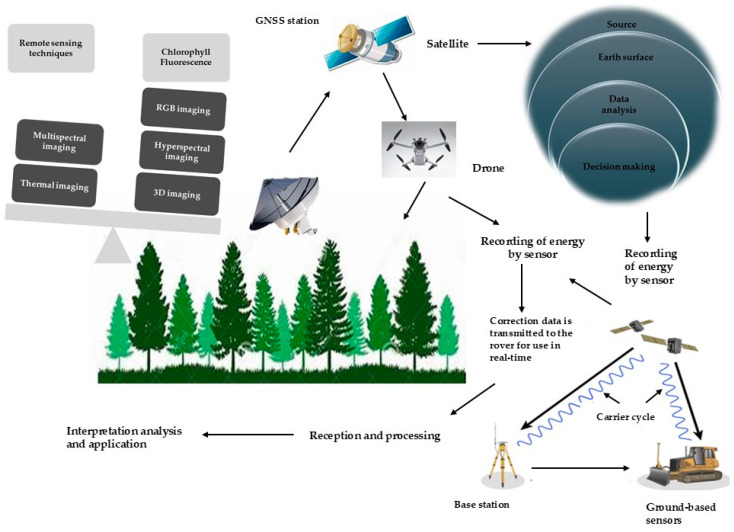
Satellite-, aircraft-, or ground-based sensors capture and record reflected or emitted energy across multiple wavelengths of the electromagnetic spectrum to remotely sense an object or area. These remote sensing techniques detect surface features, vegetation health, soil moisture, and other critical properties. The data obtained through these methods is invaluable for applications such as land use mapping, environmental monitoring, and natural resource management.

Inaccessible sensing involves observing and capturing data on target item properties without direct physical contact [125]. This method expedites nematode species identification by detecting changes in aboveground plant symptoms without damaging the host, accelerating diagnosis and reducing costs. Remote sensing techniques rapidly and non-invasively collect extensive data. Various spectroscopic and imaging methods, such as visible, multiband, infrared, and fluorescence spectroscopy, fluorescence imaging, multispectral and hyperspectral imaging, thermography, and nuclear magnetic resonance spectroscopy, have been utilized to identify plant parasitic nematodes [126]. Infrared sensors were pioneered to detect *Radopholus similis* infection in citrus trees [127]. By integrating GIS and RS, researchers accurately determined the population densities of the nematodes *G. rostochiensis* and *G. pallida* in potato crops using non-invasive hyperspectral measurements. Moreover, this comprehensive approach successfully detected and quantified populations of the nematode *Heterodera glycines* [128]. Three data preparation methods were evaluated to assess their effectiveness in detecting the presence of *H. schachtii* and *R. solanii*. Hyperspectral remote sensing technology was employed to diagnose pine wood worm disease [129,130]. A study was conducted to evaluate the effectiveness of visible light imaging, thermometry, and spectroscopy in detecting an infection caused by *H. schachtii* in two sugar beetroot varieties [131]. However, as illustrated in Figure 5, the accuracy of remote sensing methods is hindered by the misclassification of certain nematodes due to symptom overlap and insufficient survey data for developing precise nematode surveillance models.

### 10.2. Unmanned Aerial Vehicles (UAVs)

Tracking the spread of PWD using satellite remote sensing is challenging due to the scattered distribution of infected or dead pine trees. To address this issue, unmanned aerial vehicles (UAVs) captured aerial data using multi-temporal hyperspectral imaging at a spatial resolution of one meter. They utilized the Normalized Difference Vegetation Index (NDVI) and the Vegetation Index green (VIgreen) to locate fallen and discolored standing pines [132]. Researchers used the FasterRCNN deep learning framework with the RPN and Residual Neural Network (ResNet). The integration of artificial intelligence and UAV remote sensing data facilitated the creation of a PWD detection model. Fine-tuning increased the network detection accuracy to 90%. Two target detection algorithms, namely Faster-RCNN and You Only Look Once version 4 (YOLOv4), and two classic machine learning methods, random forest and support vector machine, were employed to detect diseased pine trees early using UAV remote sensing data [133].

### 10.3. Hyperspectral Imaging

Early detection remains a significant challenge in the study of forest insects and pathogens [134,135]. Defining an “early stage” can be particularly difficult in PWD infections. To address this issue, researchers have introduced the PWN into healthy pine trees, categorizing the infested trees as representative of the early stage of PWD infection [132,136]. The study employed a two-dimensional convolutional neural network (2D-CNN) to detect PWDs and extract spatial information, such as texture and color distribution, from images [137]. However, this approach cannot acquire spectral information, which is a capability of a one-dimensional CNN (1D-CNN) [138,139,140]. Hyperspectral image classification has increasingly been used in 3D-CNNs. This approach uses 1D- and 2D-CNNs to extract spectral and spatial features [141]. A 3D-CNN model utilizing HSI data has been proposed to improve PWD detection [142]. Hyperspectral approaches can determine insect infestation stages using high-resolution reflectance data. This study investigated the detection and classification of infection stages using ground hyperspectral data and examined forest biochemical components, such as chlorophyll, fresh-weight moisture, and dry-weight moisture, along with spectral continuous wavelet coefficients. Support vector machines (SVMs) were used to identify infection stages. The biochemical composition of healthy and early-stage samples was found to be similar, with spectral continuous wavelet coefficients demonstrating varying sensitivity to these subtle changes. This methodological approach enables the early detection of stress in pine forests and provides a theoretical foundation and technical recommendations for addressing future large-scale insect outbreaks. The methods can be seen in Table 1.

## 11. Fungal Species Identified for PWN Control

**Figure 6:**
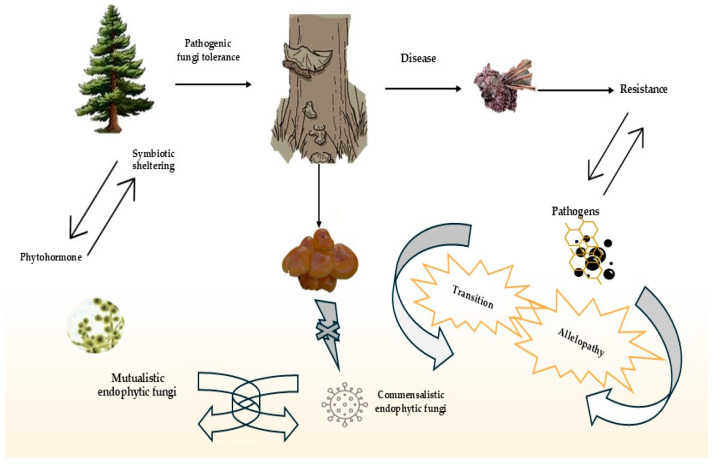
Endophytic fungi and plants collaborate through a biological pathway initiated by plant receptors detecting fungal signals. This recognition triggers a cascade of defense responses and metabolic alterations that mutually benefit both organisms, including enhanced nutrient acquisition and pathogen resistance. During this interaction, metabolites are exchanged, and gene expression is modulated in both partners, facilitating the establishment of a stable symbiotic relationship.

Since the early 1980s, studies have been conducted to document the fungal communities associated with PWD. These analyses involved cultivating fungi under different conditions, such as with the accumulation of antibiotics or using different growth media like potato dextrose agar or malt extract agar. Recently, disease-related fungal communities have been examined with fungal DNA barcode markers for molecular identification, as well as morphology and genetics [146,147]. In their research [148], Robert et al. highlighted how current standard culturing methods tend to favor fast-growing fungal species while potentially overlooking the presence of specialized fungi. They noted that our understanding of the diverse fungal population associated with complex diseases remains limited. The fungal kingdom comprises three main phyla: *Mucoromycota, Ascomycota*, and *Basidiomycota*. The dominant phylum, Ascomycota, encompasses six classes (*Blastomycetes*, *Dothideomycetes*, *Eurotiomycetes*, *Leotiomycetes*, *Orbiliomycetes*, and *Sordariomycetes*) and nearly 30 families, as shown in Table 2, which lists fungal species known to have a positive effect on PWN. It is worth noting that many of the taxa mentioned are saprophytes and might not be specific to the illness or PWN [149]. A study showed mycoflora (relationships between plant life history traits, ecology, and mycorrhizal associations) was initially isolated from symptomatic *P. thunbergii*, specifically from the shoots, twigs, and woodchips. The mycoflora was also found in *Monochamus* larvae-burrowed tunnels and pupal chambers, as well as in the adult body of *M. alternatus* after emergence. The research revealed that the genera *Ceratocystis* and *Verticicladiella* from the families *Ceratocystidaceae* and *Ophiostomataceae* were the exclusive plant species in all three examined areas. Additionally, saprophytic fungi such as Trichoderma and Penicillium were also identified. Subsequently, distinct fungi were obtained from the cerambycid beetles *M. scutellatus* and *M. carolinenses*, which were determined to be distinct from other organisms [150]. Other researchers observed that adult beetles and pupal chambers from *P. banksiana* and *P. resinosa* were frequently found in conjunction with the genera *Ceratocystis* and *Ceratocystiopsis*. Additionally, it is significant to note that the nematode-trapping fungi *Arthrobotrys cladodes* var. cladodes can be differentiated in the presence of PWN [151]. The impact of PWD on the host microbiota, however, is little understood. High-throughput sequencing in conjunction with functional prediction (FUNGuild) was used in this study to examine the fungal community and functional structures in the needles and roots of, and soil surrounding, *Pinus thunbergii* naturally infected by PWN. While no abnormalities were seen in the roots or soil, the results indicated that the fungal richness, variety, and evenness in the needles of diseased trees were considerably lower than those of healthy ones (*p* < 0.05) [152]. Maehara et al. conducted a study to examine the impact of blue-stain fungi on the population of PWNs carried by *M. alternatus* beetles emerging from logs of *P. densiflora* trees damaged by PWD. The researchers discovered that blue-stain fungi were present in 90% of pine trees affected by wilt. They also observed that the type of blue-stain fungi significantly affected the quantity of JIV nematodes carried by the beetles. Additionally, they pointed out that the quantity of nematodes found was affected by the particular pine tree and the level of moisture in the wood [153]. The study conducted by Zhao et al. demonstrated that the presence of *Sporothrix* sp. had a notable impact on the population and prevalence of the invasive PWN and native beetle symbiosis in the xylem of trees. It was found that the aromatic diacetone alcohol released by the wood infected with *Sporothrix* sp. not only boosted the reproductive ability of the nematodes, but also supported the growth and survival of the beetles [154]. Zhang et al. showed that nematode-killing fungi have developed different methods to efficiently attack nematodes, including those that are free-living and those that parasitize plants. These methods include using fungi that trap nematodes and fungi that live inside them, both of which have been useful in controlling biological populations [155]. Researchers are investigating the potential use of naturally existing fungi in separating bioactive substances that could aid in managing PWN. *Caryospora callicarpa* YMF1.01026, a freshwater fungus, generates compounds called caryospomycins, which have exhibited moderate efficacy in decreasing the PWN population owing to their nematicidal characteristics [156]. The study by Li et al. revealed the effectiveness of three substances obtained from the endophytic fungi *Geotrichum* sp. AL4, which was found on the leaves of *Azadirachta indica*, in controlling worms. Culturomics research has identified a diverse range of fungal communities, including saprophytic fungi such as *Aspergillus, Fusarium*, and *Trichoderma*, as well as necrotrophic diseases like the ophiostomatoid or blue-stain fungus associated with bark beetles. Ophiostomatoid fungi, discovered in insect pupal chambers, tunnels, and wilted pine trees, play a crucial role in the spread and proliferation of nematodes within the host tree [157]. Additionally, native microflora have been proposed as potential agents for controlling nematodes [158]. The research findings indicate that PWN infection has the potential to diminish the variety of fungi present and alter the functional makeup of the host’s microbiome. This highlights the complex relationship between PWN and fungal populations. The shift in community structure could influence the overall health of the trees and their ability to fend off nematode infestations. Furthermore, utilizing this naturally occurring fungus as a means of biocontrol presents a promising strategy for sustainable forest management. The interactions between endophytic fungi and their host plants can take various forms, including pathogenic, communalistic, and mutualistic, as illustrated in Table 2 and Figure 6.

## 12. Discussion and Future Perspectives

In this work, we have evaluated the techniques currently in use and available to diagnose PWD. The conventional detection method is employed for a preliminary PWD assessment and combined with a pathogen identification method that is both accurate and universally applicable. However, to successfully grow pine trees, it is essential to ensure they receive full sun exposure and are planted in healthy soil with a balanced pH. Fertilization and pest/disease management are critical components of plant health care, while regular light thinning helps reduce tree taper and maintain low basal area. Morphological identification remains valid, particularly when molecular approaches are impractical, although the identification can be subjective due to physical similarities with non-pathogenic species. Nematologists must possess the necessary skills to analyze minor morphological traits for accurate identification, which can be a time-consuming process, especially when isolated samples lack normal female adults, requiring separate observation of juvenile and male specimens. Controlling PWD also involves insecticide spraying to target the vector beetles that spread the pine wood nematode, although this must be used in conjunction with other strategies for effective disease management. Treatment and management of *B. xylophilus* and PWD rely significantly on natural fungal populations, with studies suggesting that some fungi, particularly ophiostomatales, exhibit nematicidal effects and may disrupt the PWN life cycle. Understanding these dynamics is crucial for developing practical biocontrol approaches, as infected and non-infected pine trees have different fungus communities. DNA-based nematode detection methods are essential in contemporary nematology, offering notable benefits compared to conventional procedures. Further research and development in this area are necessary to overcome current obstacles and enhance the effectiveness of these methods, with increased detection capabilities having the potential to significantly impact plant and animal health, environmental monitoring, and biosecurity. This underscores the importance of allocating resources to these technologies to promote sustainable agricultural practices and ecosystem management, with a comprehensive approach utilizing multiple complementary methods providing the most reliable nematode identification. EMA offers faster, more sensitive, and more specific pine wood nematode detection, with continued research potentially contributing to improved forest health monitoring and pest management. However, further improvement and standardization are required to enhance its appropriateness and dependability in various settings. Proteomics provides a powerful tool for understanding the complex details of *B. xylophilus* life and its interaction with host plants, enabling scientists to actively contribute to the development of more efficient management measures for PWD by identifying key proteins involved in virulence and comprehending their functionalities. Future research should focus on expanding the proteome database for *B. xylophilus* by including more extensive protein profiles from different isolates and environmental conditions, investigating the specific roles of identified virulence biomarkers in host–nematode interactions, and exploring the feasibility of integrating proteomic data with other molecular approaches to develop integrated pest management strategies.

**Figure 7:**
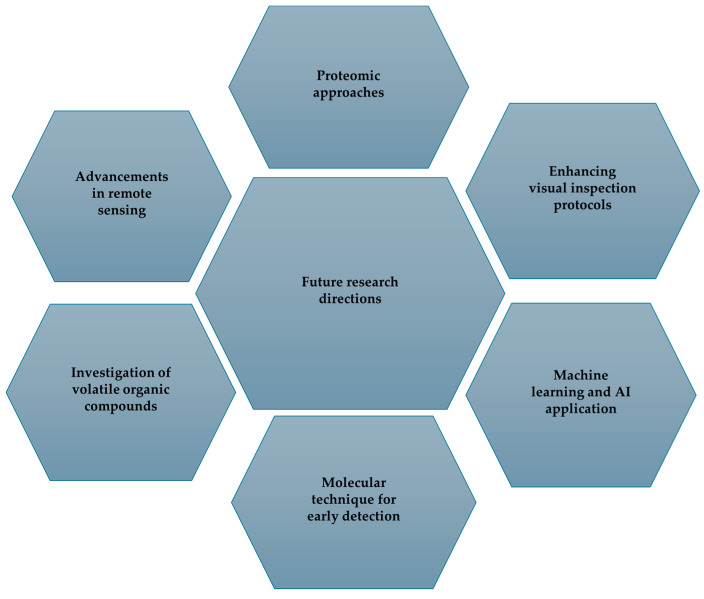
This study aims to enhance early detection methods, streamline management strategies, and mitigate the global impact of PWD on pine forest ecosystems.

Integrating remote sensing data with molecular diagnostics enables a more comprehensive assessment. Remote sensing aids in identifying potential problem areas, while PCR and sequencing are employed to conclusively determine the nematode species. Combining the benefits of remote sensing, such as rapid, wide-scale, and early detection, with the accuracy of molecular techniques, including species identification and presence confirmation, provides a robust integrated approach for nematode management. This allows for targeted and swift interventions to minimize crop damage. However, future technological advancements may facilitate the integration of multiple detection methods, thereby enhancing the ability to recognize and categorize pine wilt, as illustrated in Figure 7.

Future investigations should combine visual examination and morphological identification with advanced molecular methods such as RT-PCR, ddPCR, and rapid processes to enhance precision and understanding in diagnosing individuals with disabilities. Consistency in symptom detection can be improved through visual examination approaches and training programs. Early intervention is critical for reducing misdiagnosis rates. Further research should explore the use of UAVs equipped with advanced hyperspectral imaging technologies to detect plant water stress (PWS) across extensive areas. Multi-temporal data analysis can enhance early detection by monitoring disease progression and identifying optimal detection times. The development of rapid, field-ready molecular detection methods should be a primary focus of future research. Techniques such as LAMP-CRISPR and protein-based methods have the potential to provide swift and accurate detection of the pine wood nematode. Investigating VOC indicators in unhealthy pine trees as an early detection tool may enable non-invasive detection of asymptomatic disease. Proteome analysis of *B. xylophilus* can be conducted to identify protein markers associated with infection, aiding in species identification and understanding pathogen–host interactions. Applying machine learning algorithms to analyze UAV and hyperspectral data can improve the detection of PWS. To facilitate timely interventions, artificial intelligence algorithms must accurately diagnose disease stages based on spectral characteristics.

## Figures and Tables

**Table 1:** Advantages and disadvantages of PWD detection methods.

Method	Advantages	Disadvantages	Reference
Visual tree survey			
Ground survey	Lightweight and easy to work	Necessitates professional experience	[143]
DNA-based techniques			
RFLP	Codominant markers, distinguishes the heterozygote	Radioactive probes, complex, time-consuming	[53,61,62]
RAPD	Quick and easy to assay	Low level of polymorphism, dominant mode, low reliability	[50,54]
ALFP	Great genomic abundance	Accuracy in fragment dimension, less ideal replication	[65,66]
DNA barcoding	Robust amplification system, convenient fragment sizes,	Time-consuming	[144]
Microarray method	Versatility, specificity, speed	High cost, difficulty in distinguishing	[78]
Sequence-based method	Ability to discover genetic variants, wide rand	More expensive, longer time,	[91]
Rapid method	Speed, ability to detect pathogens, high sensitivity	Risk of contamination, high cost	[92]
Protein-based techniques			
Gel Electrophoresis	Simplicity, visual results, purification	Time-consuming, sample loss	[108]
Isozyme method	Stable markers, non-destructive, beneficial for a breeding program	Less information, tissue specificity	[111]
RT-PCR	Rapid setup, affordability, lack of contamination	Test is too complex, and tools are relatively expensive	[63]
ddPCR	Simple, convenient, high sensitivity	Expensive instruments	[104]
LAMP-CRISPR/Cas12a	High sensitivity and specificity, isothermal amplification	Potential for false positives, optimization challenges	[117]
EMA	Fast, accurate detection	Optimization requirements	[119]
Digital Methods			
Satellite detection	Significant area detection	It is challenging to document specific changes	[54]
UAVs	Significant area detection	Requires technological organization expertise	[145]
Hyperspectral descriptions	Color distribution and texture	High cost and complexity	[137]

**Table 2:** List of fungi–PWN interactions.

PWN Interaction with Fungi	Activity	Reference
*Alternaria* sp. *Epicoccum* sp.	A reduction in the development of PWN in mycelial fungus grown in vitro	[159]
*Aureobasidium* sp. *Aspergillus* sp. *Gliocladium* sp. *Mucor* sp. *Mortierella* sp. *Penicillium* sp. *Rhizoctonia* sp.	A reduction in the development of PWN in mycelial fungus grown in vitro	[158]
*Cystidiophorus castaneus*	*Pinus densiflora* PWN population growth decreases; decrease in developing *Monochamus alternatus* PWNs.	[158]
*Cephalosporium* sp.	A reduction in the development of PWN in mycelial fungus grown in vitro	[160]
*Fusarium* sp.	A reduction in the development of PWN in mycelial fungus grown in vitro	[161]
*Pycnoporus coccineus*	Population growth decreases; a decrease in developing *Monochamus alternatus* PWNs.	[162]
*Trichoderma* sp.	In vitro mycelial fungi *Pinus densiflora* and *P. thunbergii* wood blocks show decreased PWN population expansion; emergent *Monochamus alternatus* carries fewer PWNs.	[158]
*Caryospora callicarpa*	PWN is moderately killed by caryospomycins A to C metabolites.	[156]

## Data Availability

Data are contained within the article.
